# “Nurses and midwives: clean care is in your hands”: the 5th May 2020 World Health Organization *SAVE LIVES: Clean Your Hands* campaign

**DOI:** 10.1186/s13756-020-00717-6

**Published:** 2020-04-19

**Authors:** Alexandra Peters, Nasim Lotfinejad, Chloé Guitart, Alice Simniceanu, Maria Clara Padoveze, Tcheun Borzykowski, Benedetta Allegranzi, Didier Pittet

**Affiliations:** 1 Infection Control Program, University of Geneva Hospitals and Faculty of Medicine, Geneva, Switzerland; 2 Department of Research, Faculty of Medicine, Mashhad University of Medical Sciences, Mashhad, Iran; 3 Infection Prevention and Control Hub and Antimicrobial Resistance Division, World Health Organization, Geneva, Switzerland; 4 Infection Prevention and Control Hub, Integrated Health Services, UHC/Life Course, World Health Organization, Geneva, Switzerland

**Keywords:** Infection prevention and control, Infection control, Hand hygiene, Nurses, Midwives, World Health Organization, Healthcare-associated infection

## Abstract

In honor of Florence Nightingale’s 200th birthday, the World Health Organization (WHO) has declared 2020 the “Year of the Nurse and Midwife”. On May 5th of this year, for the annual celebration of the SAVE LIVES: Clean Your Hands campaign, WHO will focus on the essential role that nurses and midwives play in contributing to saving millions of lives per year. It is necessary to recognize the work and the immense responsibility that nurses and midwives carry since achieving Universal Health Coverage is highly reliant on them.

## Background

In honor of Florence Nightingale’s 200th birthday, the World Health Organization (WHO) has declared 2020 the “Year of the Nurse and Midwife”. In addition to championing the nursing profession, Nightingale’s role was also fundamental for the recognition of the importance of infection prevention and control (IPC), as she was among the first to recognize that a caregiver could transmit germs, and thus cause patient harm. Nurses and midwives make up nearly 50% of the global health workforce [1], and are the group of healthcare workers that have the most frequent contact with patients. This makes them pivotal figures in the fight against healthcare-associated infections (HAI) as well as neonatal and maternal sepsis.

**Fig. 1:**
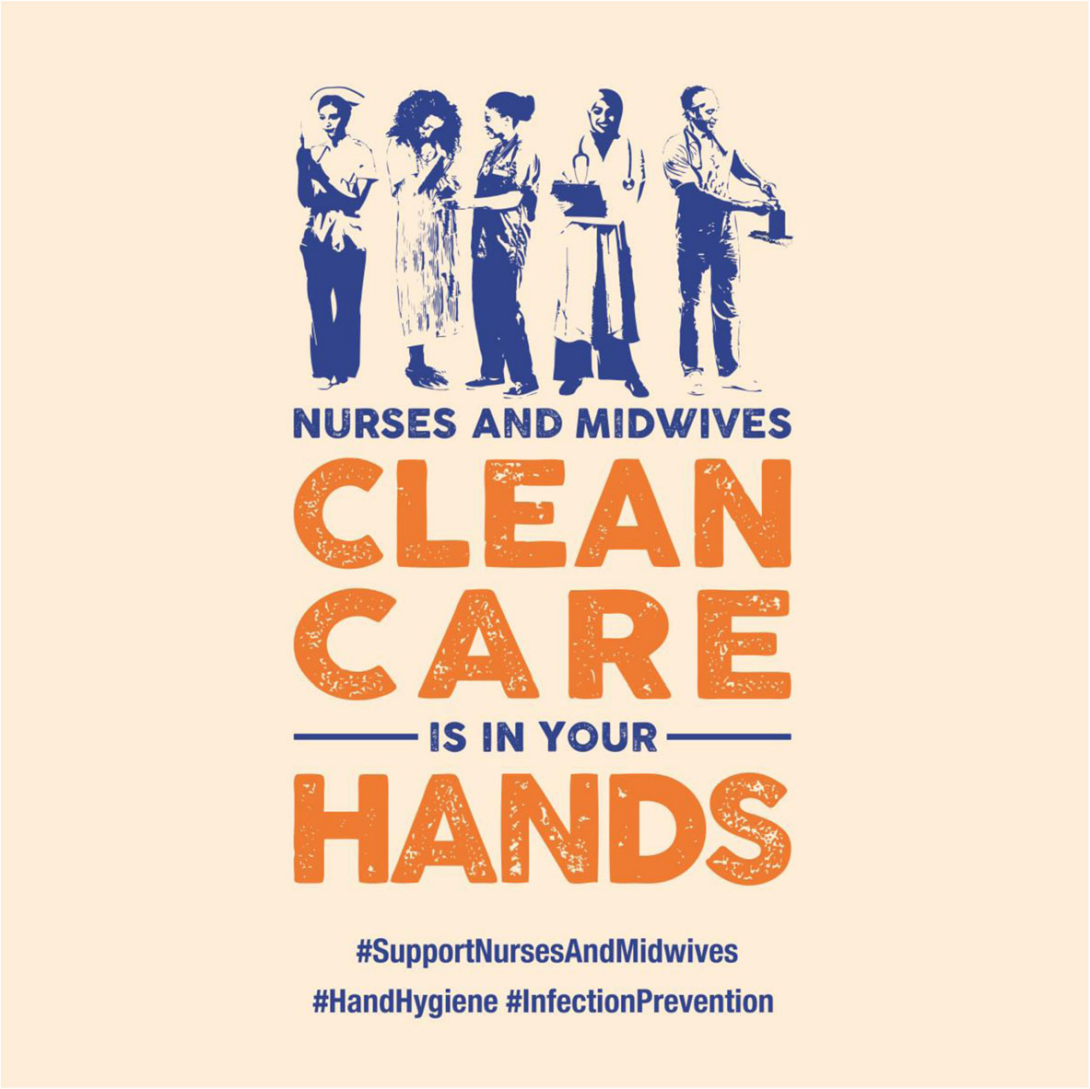
May 5, 2020: “Nurses and Midwives, clean care is in your hands!”. The May 5, 2020, World Health Organization SAVE LIVES: Clean Your Hands campaign slogan and main promotional image (2020 hashtags: #SupportNursesAndMidwives #HandHygiene #InfectionPrevention). Campaign participants are invited to submit photos or selfies of them holding a board with the slogan and hashtags at www.CleanHandsSaveLives.org

On the 5th May 2020, for the annual celebration of the *SAVE LIVES: Clean Your Hands* campaign, WHO will focus on the essential role that nurses and midwives play in contributing to saving millions of lives each year by championing clean care (Fig. 1). Despite many improvements around the world, rates of HAI remain unacceptably high, and the majority of them are transmitted by healthcare workers’ hands. Therefore, hand hygiene promotion strategies must be constantly reinforced and improved. Clean healthcare has recently been recognized by WHO as one of the most urgent challenges to be tackled by the global community over the next ten year [2]. Actively engaging the expertise of nurses and midwives in the development, implementation and evaluation of hand hygiene promotion contributes to clean healthcare.

Along with recognizing the critical importance of nurses and midwives to patient care, the aim of the “Year of the Nurse and Midwife” is also to highlight that there is a major global shortage of healthcare workers, and that more than half of the shortage is of nurses and midwives [1]. WHO estimates that for countries to succeed in reaching the Sustainable Development Goal # 3 on health and well-being, the world will need an additional 9 million nurses and midwives by the year 2030 [1]. It has been proven that investing in education and job creation in the health and social sectors will result in improved health outcomes, global health security, and economic growth [1]. Having adequate healthcare worker staffing reduces the risk of HAI and antimicrobial resistance, and is thus recommended by WHO as a core component of effective IPC programmes [3].

It is crucial to recognize both the work and the immense responsibility that nurses and midwives carry: we cannot achieve Universal Health Coverage without investing in them. Everyone- including policy makers, healthcare workers, and patients themselves- can contribute to improving hand hygiene and preventing infections (Table 1).

**Table 1:** The 5 May 2020 World Health Organization *SAVE LIVES: Clean Your Hands* Campaign Calls to Action

Campaign Participants	Call to Action
Nurses	“Clean and safe care starts with you.”
Midwives	“Your hands make all the difference for mothers and babies.”
IPC leaders	“Empower nurses and midwives in providing clean care.”
Policy makers	“Increase nurse staffing levels to prevent infections and improve quality of care. Create the means to empower nurses and midwives.”
Patients and families	“Safer care for you, with you.”

Abbreviations: *IPC* infection prevention and control; *WHO* World Health Organization

Please join us in celebrating this vital and often underappreciated group of HCW; “Nurses and Midwives: CLEAN CARE is in YOUR HANDS”!

## Conclusion

Nurses and midwives make are the group of healthcare workers that have the most frequent contact with patients, making them pivotal figures in the fight against healthcare- associated infections as well as neonatal and maternal sepsis. Despite many improvements around the world, rates of healthcare- associated infections remain unacceptably high, and the majority of them are transmitted by healthcare workers’ hands. Please join us in celebrating this vital and often underappreciated group of healthcare workers. “Nurses and midwives: CLEAN CARE is in YOUR HANDS”!

## Data Availability

Data sharing not applicable to this article as no datasets were generated or analyzed during the current study.
